# Commencement Southern Medical College

**Published:** 1893-03

**Authors:** 


### THE FOURTEENTH ANNUAL COMMENCEMENT OF THE SOUTHERN MEDICAL COLLEGE.

The closing exercises of the Southern Medical College occurred at DeGive's opera house, on the evening of March 2d, in the presence of a very large audience.

The members of the graduating classes in both the medical and dental departments of the institution occupied seats in front of the stage.

The institution known as the Southern Medical College embraces four departments, the medical, dental, pharmaceutical and law departments, all of which are in a thriving and healthy condition.

The exercises were opened with prayer by the Rev. A. R. Holderby.

After the report of the dean the diplomas of the graduating classes were awarded, thirty-seven in the medical department.

The degree of "doctor of medicine" was first conferred upon the following gentlemen:

George O. Allen, Harry M. Clark, William B. Brown, Alza B. Claughton, John R. S. Carr, Thomas Z. Clower, James D. Creamer, Colonel W. Corbett, Frank L. Dennis, Joseph Corbett, George A. Dennis, James McF. Gaston, Jr., Charles F. Fickling, Vatia D. Gay, Jesse O. Gray, William C. Hathcock, Waymon T. Gunter, Joseph B. Hunter, Thomas Hall, James W. Long, Ambrose, W. Martin, John T. Longino, Jr., Joseph L. Murdock, Charles F. Maddox, James E. Overstreet, Reuben H. Poole, David H. Perry, Lyman U. Rentz, George R. Plummer, Luke Robinson, George B. Sharpe, James A. Sewell, Andrew Temples, John R. Shannon. John W. Watts, Carl C. Wilson, Jr., George T. Williamson.

After the diplomas were conferred, the valedictorian of the medical department, Dr. John R. Shannon, of Georgia, was then introduced.

The next speaker was the valedictorian of the dental department, Dr. R. A. Caswell, of Florida.

The last feature of the exercises was the award of medals.

In the medical department, the first honor was shared by three gentlemen, who received as their reward three medals. These gentlemen were Drs J. McF. Gaston, Jr., of Atlanta,; D. H. Perry, of Florida; and F. L. Dennis, of Atlantta.

The second honor was awarded to Dr. W. C. Hancock, of Atlanta, Ga., and the third to Dr. G. R. Plummer, of Key West, Fla.

The Crichton medal was awarded to Dr. F. L. Dennis.

In the dental department, six medals were awarded. The first to Dr. W. R. Tyler, of Atlanta, and the second to Dr. E. C. Smith, of South Carolina.

The Crenshaw medal was awarded to Dr. E. C. Smith, of South Carolina; the McRae medal to Dr. Bryan, of West Virginia; the Brown medal to Dr. G. T. Williamson, and the Jewet medal to Dr. S. A. King, of Texas.

After the graduating exercises, the annual banquet was held at the Kimball House.

